# Correction to: Long-term Safety and Tolerability of Omadacycline for the Treatment of *Mycobacterium abscessus* Infections

**DOI:** 10.1093/ofid/ofad446

**Published:** 2023-08-22

In the original publication of this article [Mingora CM, Bullington W, Faasuamalie EP et al. Long-term Safety and Tolerability of Omadacycline for the Treatment of *Mycobacterium abscessus* Infections. Open Forum Infectious Diseases, Volume 10, Issue 7, August 2023, ofad335, https://doi.org/10.1093/ofid/ofad335], the author Ryan Stadnik's name was incorrectly spelled as ‘Ryan Stadnick’. This has been corrected in the version of the article currently available online.

